# Studies on bacterial community composition are affected by the time and storage method of the rumen content

**DOI:** 10.1371/journal.pone.0176701

**Published:** 2017-04-28

**Authors:** Yury Tatiana Granja-Salcedo, Ricardo Andrés Ramirez-Uscategui, Elwi Guillermo Machado, Juliana Duarte Messana, Luciano Takeshi Kishi, Ana Veronica Lino Dias, Telma Teresinha Berchielli

**Affiliations:** 1 Department of Animal Science, Faculdade de Ciências Agrárias e Veterinárias (FCAV), UNESP - Univ Estadual Paulista, Jaboticabal, São Paulo, Brazil; 2 Department of Clinical and Veterinary Surgery, Faculdade de Ciências Agrárias e Veterinárias (FCAV), UNESP - Univ Estadual Paulista, Jaboticabal, São Paulo, Brazil; 3 Grupo de Investigación en Medicina Genómica - GIMEGEN, Universidad Simón Bolívar, Barranquilla, Colombia; 4 Department of Technology, Faculdade de Ciências Agrárias e Veterinárias (FCAV), UNESP - Univ Estadual Paulista, Jaboticabal, São Paulo, Brazil; 5 Department of Animal Science, INCT/CA – Universidade Federal de Viçosa (UFV), Viçosa, Minas Gerais, Brazil; Wageningen University, NETHERLANDS

## Abstract

The objective of this study was to investigate three storage methods and four storage times for rumen sampling in terms of quality and yield of extracted metagenomic DNA as well as the composition of the rumen bacterial community. One Nellore steer fitted with a ruminal silicone-type cannula was used as a donor of ruminal contents. The experiment comprised 11 experimental groups: pellet control (PC), lyophilized control (LC), P-20: pellet stored frozen at -20°C for a period of 3, 6, and 12 months, P-80: pellet stored frozen at -80°C for a period of 3, 6, and 12 months, and L-20: lyophilized sample stored frozen at -20°C for a period of 3, 6, and 12 months. Metagenomic DNA concentrations were measured spectrophotometrically and fluorometrically and ion torrent sequencing was used to assess the bacterial community composition. The L-20 method could not maintain the yield of DNA during storage. In addition, the P-80 group showed a greater yield of metagenomic DNA than the other groups after 6 months of storage. Rumen samples stored as pellets (P-20 and P-80) resulted in lower richness Chao 1, ACE, and Shannon Wiener indices when compared to PC, while LC and PC were only different in richness ACE. The storage method and storage time influenced the proportions of 14 of 17 phyla identified by sequencing. In the P-20 group, the proportion of Cyanobacteria, Elusimicrobia, Fibrobacteres, Lentisphaerae, Proteobacteria, and Spirochaetes phyla identified was lower than 1%. In the P-80 group, there was an increase in the proportion of the Bacteroidetes phylum (p = 0.010); however, the proportion of Actinobacteria, Chloroflexi, SR1, Synergistetes, TM7, and WPS.2 phyla were unchanged compared to the PC group (p > 0.05). The class Clostridium was the most abundant in all stored groups and increased in its proportion, especially in the L-20 group. The rumen sample storage time significantly reduced the yield of metagenomic DNA extracted. Therefore, the storage method can influence the abundance of phyla, classes, and bacterial families studied in rumen samples and affect the richness and diversity index.

## Introduction

Ruminants have a complex digestive system, wherein the rumen microbial community plays important roles in host animal health, productivity, and the environment [1]. Bacterial populations constituting the higher microbial biomass population are the most active and fermentative and are considered the most important nutritional component in the rumen microbiome [2].

Rumen microbial ecosystem studies require new gene-based technologies that overcome the limitations of culture-based techniques, which provide the accurate and rapid enumeration of microbial populations. These are essential tools to increase the knowledge related to ruminal microbial diversity, and to better understand the roles of these microorganisms in the conversion of feed into end products [3–4].

Several studies have demonstrated that the representative rumen microbial community can be influenced by the DNA extraction method [5–7]. It can also be affected by the sampling technique of rumen contents and the fractionation of the rumen sample; in addition, sample handling and collection times can affect the results of bacterial composition studies [6, 8–12].

However, nutritional assays require several experimental time points; in addition, specific equipment and kits are required for the extraction and quantification of metagenomic DNA from the rumen sample. Thus, in some places where these technologies are emerging, methods for the storage of ruminal samples for microbiological analyses, occasionally for several months depending on experimental conditions and financial resources, are required. To date, there are no standard rumen sample storage methods used before DNA extraction; however, the most commonly used among these methods are lyophilisation [6, 13, 14], freezing at -80°C [2, 7, 15–18] or at -20°C [19–22], and the storage of rumen microbial samples as pellets with the use of buffer solutions [23–28]. However, previously mentioned studies did not refer to the ruminal sample storage time before the extraction of metagenomic DNA. Thus, great variation in storage methods can lead to uncertainty regarding the effect of these techniques on the microbial community structure and the possible bias of published results.

Accordingly, the hypothesis of this study was that the storage method and storage time could influence the yield and purity of metagenomic DNA obtained and can modify the representative rumen bacterial composition. Therefore, the objective of this study was to determine the possible effects of three storage methods and four storage times on purity and the yield of extracted metagenomic DNA as well as the composition of the rumen bacterial community.

## Materials and methods

This study was carried out in strict accordance with the recommendations in the Brazilian College of Animal Experimentation (COBEA—Colégio Brasileiro de Experimentação Animal) guidelines. The protocol was approved by the Ethics, Bioethics, and Animal Welfare Committee (CEBEA—Comissão de Ética e Bem Estar Animal) of Facultade de Ciencias Agrarias e Veterinarias (FCAV) of the Universidade Estadual Paulista (UNESP), Jaboticabal campus, Brazil (Protocol number 07784/14). Ruminal cannulation was conducted 8 months prior to the experiment under xylazine sedation and local anaesthesia with lidocaine hydrochloride, and all efforts were made to minimize suffering.

### Rumen sampling and storage method

One Nellore steer (383 kg body weight) fitted with a ruminal silicone-type cannula (diameter 10 cm) was used as a donor of ruminal contents. The animal was maintained grazing an individual paddock (*Brachiaria brizantha ‘Marandu’*) for 30 days with free access to water and a mineral mixture (containing per kg: 146 g calcium, 40 g phosphorus, 40 g sulphur, 130 g sodium, 1.35 g copper, 1.04 g manganese, 5 g zinc, 100 mg iodine, 80 mg cobalt, 26 mg selenium; maximum of 800 mg fluor).

Samples (a mix of liquid and solid) from the dorsal, central, and ventral regions of the rumen were collected through the rumen cannula to form one composite sample (approximately 4 kg). Rumen samples were placed into a thermo box cooled to 4°C and transferred to the laboratory within 15 min.

The rumen samples were homogenized and divided into sixty-six sub-samples of approximately 60 g each, which were randomly distributed into 11 experimental groups (six repetitions each) as follows. The pellet control (PC) consisted of 60 g of the rumen contents, which were immediately added to 60 ml of phosphate saline buffer (pH 7.4), stirred vigorously for 3 min and then filtered with a mesh fabric (100 μm). The filtrate was subjected to centrifugation at 16,000 × *g* for 10 min at 4°C. The supernatant was discarded and the remaining pellet was resuspended in 4 ml of tris-EDTA buffer (10×, pH 8.0). The resuspended content was centrifuged at 16,000 × *g* for 10 min at 4°C, and the supernatant was discarded. For the lyophilized control (LC), 60 g of the rumen contents were weighed, immediately frozen at -80°C during 24 h, and lyophilized for 72 h in a ModulyoD Freeze Dryer (Thermo Electron Corporation, Nepean, ON, Canada). For the P-20 group, the pellet was stored frozen at -20°C for a period of 3, 6, and 12 months. For the P-80 group, the pellet was stored frozen at -80°C for a period of 3, 6, and 12 months. For the L-20 group, the lyophilized sample was stored frozen at -20°C for a period of 3, 6 and 12 months.

### Metagenomic DNA extraction

A ‘Fast DNA SPIN Kit for Soil’ (MP Bio^®^, Biomedicals, Illkirch, France) extraction kit was used to extract metagenomic DNA from 200 mg of sample according to the manufacturer’s instructions. For PC and LC groups, the metagenomic DNA extraction was performed immediately after obtaining the pellet or after lyophilisation, respectively. In the other groups, metagenomic DNA extraction was performed at the indicated storage time for each group (3, 6, or 12 months).

All rumen samples were analysed for dry matter (DM) by drying at 105°C for 24 h to express the apparent specific metagenomic DNA yield as mg of DNA per dry weight of rumen of sample. Metagenomic DNA concentrations were measured spectrophotometrically (NanoDrop^®^ ND-1000 Spectrophotometer, Thermo Fisher Scientific, Waltham, MA, USA) and fluorometrically (Qubit^®^ 3.0, kit Qubit^®^ dsDNA Broad Range Assay Kit, Life Technologies, Carlsbad, CA, USA). The purity of metagenomic DNA was assessed spectrophotometrically at A260/A230 nm and A260/A280 nm ratios were obtained to indicate contamination of DNA with buffer salts and organic compounds. Integrity was determined by agarose gel electrophoresis using a 0.5% (wt/vol) gel, and subsequent staining with ethidium bromide (5 mg/ml).

### Amplification and sequencing

Metagenomic DNA samples of similar yield and quality were considered to select three subsamples of PC, LC, 12-month stored P-20, 12-month stored P-80, and 12-month stored L-20 groups, for assessment of the bacterial community composition based on gene sequencing of the V4 region of 16S rRNA.

Primers for PCR and sequencing that were used in this analysis were described by Caporaso et al. [29]. Each sample was amplified in triplicate, and each PCR reaction mixture (20 μl final volume) contained 20 ng of metagenomic DNA, 10 μM of each forward and reverse primers, 1.25 mM of magnesium chloride, 200 μM of dNTP mix (Invitrogen, Carlsbad, CA, USA), 1.0 U platinum Taq DNA polymerase high fidelity (Invitrogen, Carlsbad, CA, USA), high fidelity PCR buffer [1X], and milli-Q water. Reactions were held at 95°C for 3 min to denature the DNA, with amplification proceeding for 40 cycles at 95°C for 30 s, 53.8°C for 30 s, and 72°C for 45 s; a final extension of 10 min at 72°C was added to ensure complete amplification.

The expected fragment length of PCR products was verified by agarose gel (1%) electrophoresis and the amplicon size was estimated by comparison with a 1 kb plus DNA ladder (1 kb plus DNA ladder, Invitrogen, Carlsbad, CA, USA). The PCR fragments were purified using the Zymoclean^™^ Gel DNA Recovery kit following the manufacturer's instructions. Composite samples for sequencing were created by combining equimolar ratios of amplicons from the triplicate samples. Sequencing was performed using the Ion Torrent Personal Genome Machine (Life Technologies, Carlsbad, CA, USA) using the Ion 314^™^ Chip Kit v2 at the Sequencing Facility, Department of Technology of FCAV, Jaboticabal, Brazil.

### Data analysis

Sequence data were processed removing adapter using Scythe 0.991 (https://github.com/vsbuffalo/scythe) and Cutadapt 1.7.1 [30]. Sequence trimming was carried out by selecting sequences over 200 bp in length with an average quality score greater than 20 based on Phred quality and duplicate reads were removed using the Prinseq program [31]. We used the Quantitative Insights Into Microbial Ecology (QIIME) software package version 1.9.1 to filter reads and determine Operational Taxonomic Units (OTUs) as described in Caporaso et al. [32]. The Usearch algorithm was used to cluster the reads OTUs with a 97% cutoff, and to assign the taxonomy using the Ribosomal Database Project (RDPII) version 10 [33]. Bacterial sequences were de-noised and suspected chimeras were removed using the OTUpipe function within QIIME. Sequence data were were summarized at the phylum, class, and family levels; in addition, Alpha_diversity.py in QIIME was used to calculate ACE, Chao1, Shannon and Simpson indices. Principal coordinate analysis (PCoA) was conducted to evaluate differences in community structure among experimental groups (β-diversity). PCoA was generated using unweighted Unifrac distance [34] using the R package vegan version 2.0–10.

Statistical analyses were performed with the assistance of R software [35]. Data of metagenomic DNA yield and purity were compared between storage method and storage time of rumen samples using a Friedman’s test and a Dunn’s post-hoc test. Bacterial community composition at different phylogenetic levels was compared between experimental groups using a Kruskal-Wallis test and a Dunn’s post-hoc test. A probability of p < 0.05 was considered significant for all tests.

## Results

### Metagenomic DNA yield and purity

**Fig 1:**
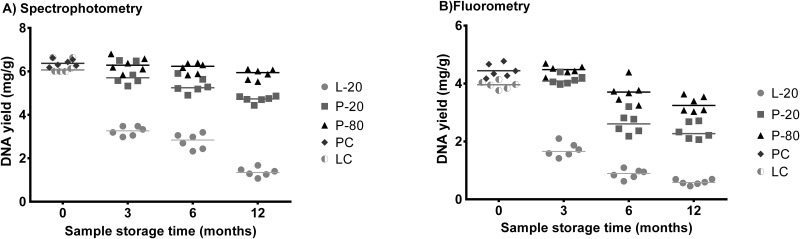
Apparent specific metagenomic DNA yield (mg g^-1^ dry weight rumen contents) quantified by spectrophotometry (A) or fluorometry (B) of three methods and four storage times of ruminal samples. PC = pellet control, LC = lyophilized control, P-20 = pellet stored frozen at -20°C; P-80 = pellet stored frozen at -80°C; L-20 = lyophilized sample stored frozen at -20°C.

The specific yield (mg DNA/g sample) of metagenomic DNA varied, depending on the sample storage time and rumen sample storage method used when both were quantified by spectrophotometry (Fig 1A; p < 0.001) or fluorometry (Fig 1B; p < 0.001). Thus, when the rumen samples were not stored (PC and LC groups) no effects of the sample storage method were observed (p > 0.05). However, after 3 months of storage, both pellet sample groups (P-80 and P-20) showed higher yield than the lyophilized samples at -20°C (L-20). In addition, the P-80 group showed a greater yield of metagenomic DNA than the other groups after 6 months of storage.

Therefore, the L -20 storage method cannot maintain the yield of DNA. At 12 months of storage for the L-20 group 21.83% of DNA extracted from the LC group was recovered, resulting in metagenomic DNA losses of approximately 6.5% per month of rumen sample storage. Moreover, 73.83% of DNA was recovered from the P-20 group and the P-80 group resulted in a high (p < 0.001) recovery of 91.83% of DNA obtained from the PC group.

The relative absorbance readings for A 260/A280 nm and A 260/A230 nm (1.89 ± 0.08 and 1.95 ± 0.19 median ± IQR, respectively) were not affected by the time or rumen sample storage method (p > 0.05). DNA integrity determined by agarose gel apparently was not affected when the rumen samples were not stored (S1 Fig). However, after 6 months of storage, all methods showed poor DNA integrity, especially lyophilized samples.

### Sequences

Ion Torrent sequencing produced 1,131,462 sequences from the 15 samples. The number of the generated sequences was not affected by the time or rumen sample storage method (p > 0.05). The median number of sequences was 76,105 per sample and the average length of the quality sequences was 354 bp. All rarefaction curves for each sample approached the saturation plateau (S2 Fig).

The PC group led to the highest richness of the rumen bacterial population (Table 1), characterized by the highest number of observed species and the highest value of ACE and Chao 1 (p < 0.05) when compared to P-20 and P-80 groups, while LC and PC were only different in richness ACE (p = 0.017). Meanwhile, the lyophilized groups presented lower OTUs remaining after the pipeline (lyophilized 66% vs pellet 71%).

**Table 1:** Median and interquartile range of the number of observed operational taxonomic units (OTUs), richness (Chao1 and ACE) and diversity estimators (Shannon Wiener and Simpson) in pellet control (PC), pellet stored frozen at -20°C (P-20), pellet stored frozen at -80°C (P-80), lyophilized control (LC) and lyophilized sample stored frozen at -20°C (L-20) groups.

	PC	P-20	P-80	LC	L-20	p-value
OTUs	1241 ± 6^a^	1174 ± 14^c^	1187 ± 12^b^	1203 ± 8^a^	1210 ± 28^a^	0.017
Chao 1	1272 ± 5^a^	1206 ± 17^b^	1211 ± 13^b^	1249 ± 21^a^	1214 ± 32^b^	0.030
ACE	1264 ± 6^a^	1195 ± 13^b^	1207 ± 9^b^	1225 ± 14^b^^c^	1237 ± 26^a^	0.017
Shannon Wiener	8.47 ± 0.1^a^	8.07 ± 0.1^b^	8.18 ± 0.1^b^	8.58 ± 0.1^a^	8.53 ± 0.1^a^	0.001
Simpson	0.994 ± 0.1	0.988 ± 0.1	0.989 ± 0.1	0.993 ± 0.1	0.993 ± 0.1	0.281

^a, b, c, d^ = values followed with superscript letters indicate statistical differences (P < 0.05) based on Dunn’s test.

### Bacterial community composition

**Fig 2:**
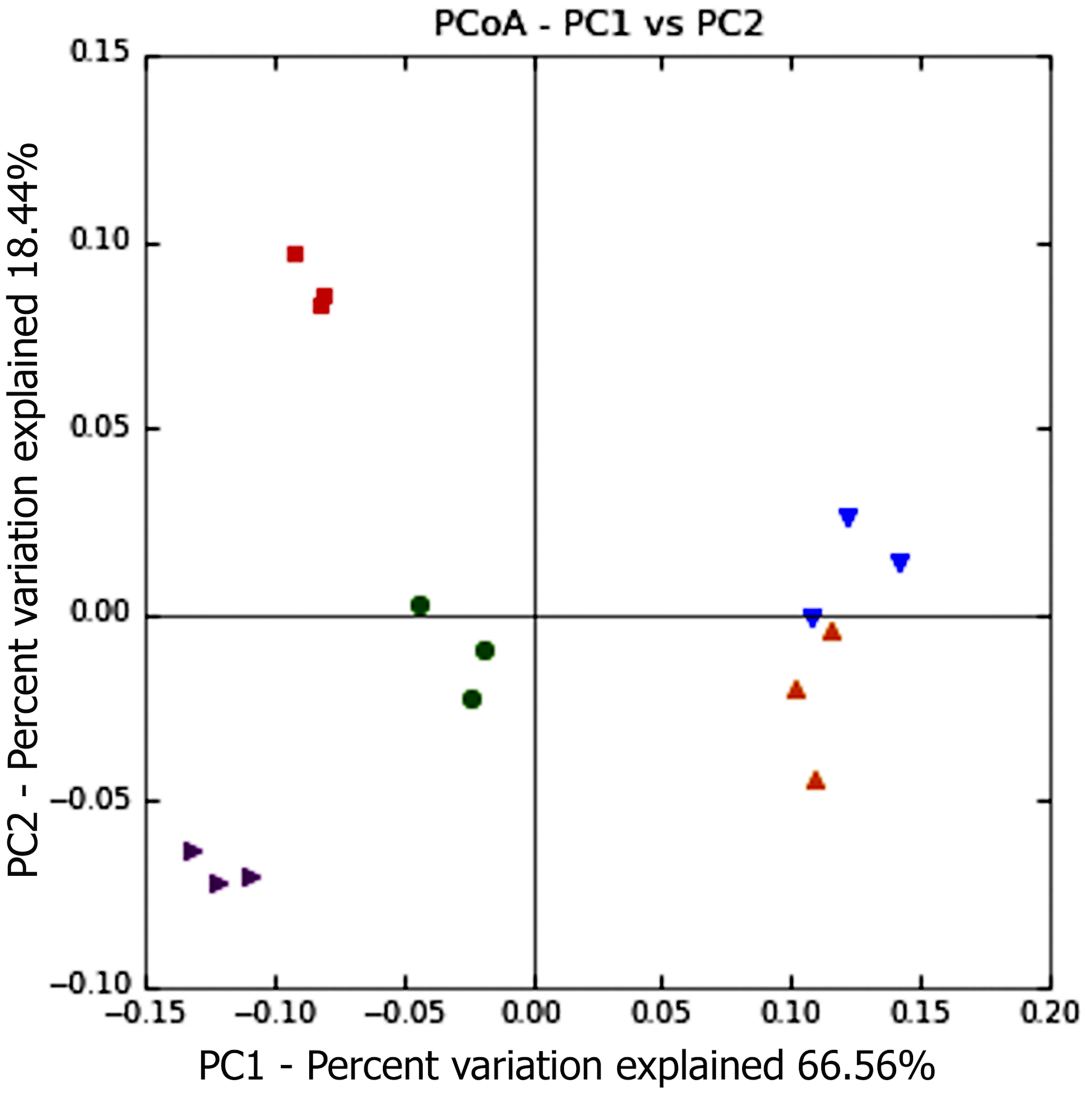
Weighted Unifrac diversity principal coordinate analysis (PCoA) to explore dissimilarities in microbial composition among pellet control (purple triangle), pellet stored frozen at -20°C (blue triangle) pellet stored frozen at -80°C (orange triangle), lyophilized control (green circle) and lyophilized sample stored frozen at -20°C (red square) groups.

The comparison of the bacterial communities by principal coordinate analysis (PCoA) using the weighted Unifrac distance (Fig 2), explains 85% of the variation in the data and confirmed a separation between PC group and both pellet sample groups (P-80 and P-20), while the L -20 storage group was more distant from another groups.

**Fig 3:**
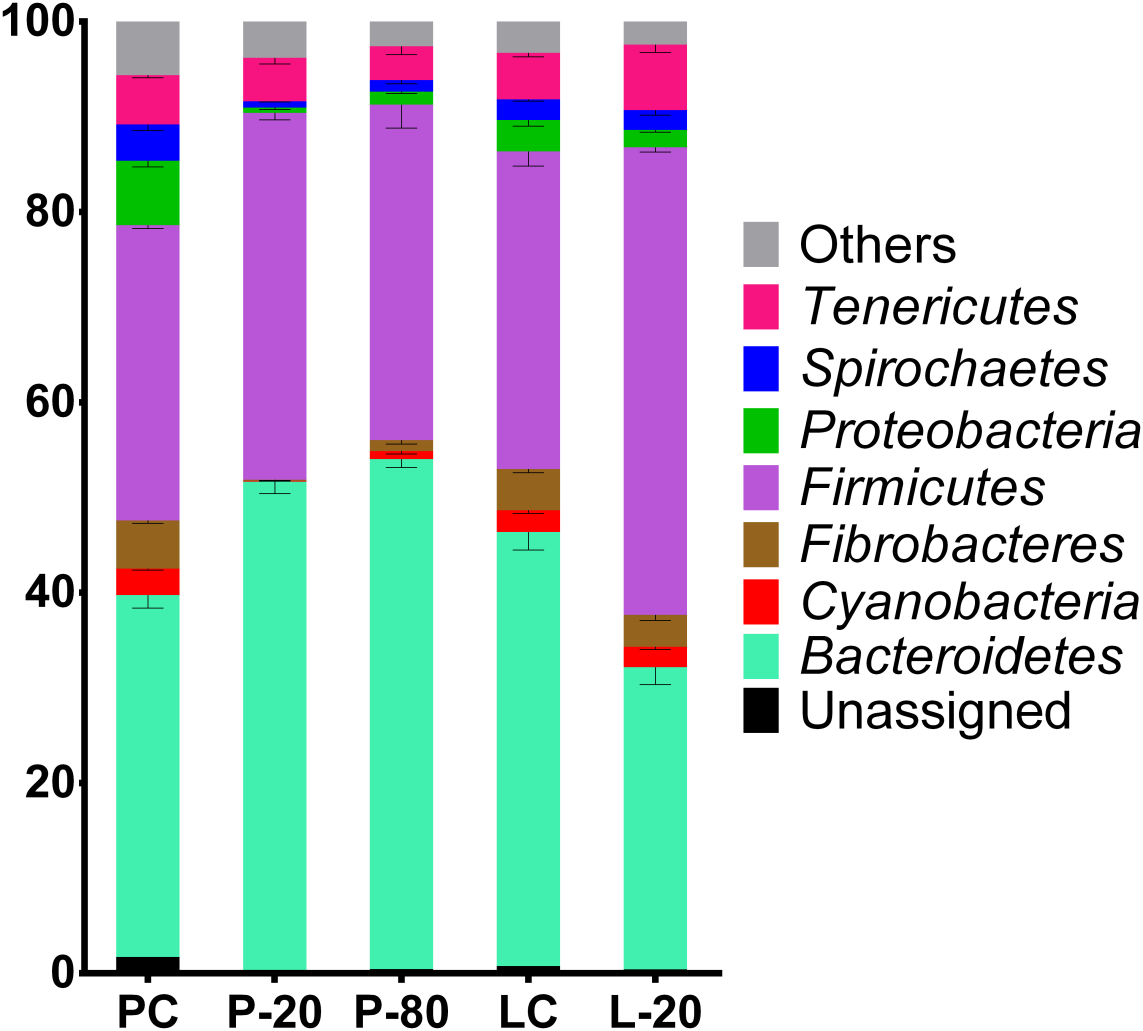
Abundance of operational taxonomic units (%) classified at the phylum level in pellet control (PC), pellet stored frozen at -20°C (P-20) pellet stored frozen at -80°C (P-80), lyophilized control (LC) and lyophilized sample stored frozen at -20°C (L-20) groups. Others = sum of Actinobacteria, Chloroflexi, Elusimicrobia, Lentisphaerae, Planctomycetes, SR1, Synergistetes, TM7, Verrucomicrobia, WPS-2, Fusobacteria, and Armatimonadetes. n = 3 samples per group. P-20, P-80 and L-20 groups were stored for 12 months.

Sequencing the V4 region of 16S rRNA gene allowed for the detection of 17 phyla in all groups (S1 Table). Bacteroidetes, Firmicutes, Fibrobacteres, Tenericutes, and *Cyanobacteria* phyla were the most abundant in all samples and accounted for over 88% of the total bacterial community (Fig 3). Storage method and storage time showed an effect on the relative abundance of 14 of the 17 phyla detected by sequencing. Only SR1 (0.46%), TM7 (0.14%), and Synergistetes (0.05%) phyla were not influenced by sample storage time and rumen sample storage method (p > 0.05).

The PC group presented a higher abundance of Cyanobacteria, Fibrobacteres, Lentisphaerae, Proteobacteria, and Spirochaetes and a lower abundance of Bacteroidetes, Chloroflexi, and Firmicutes phyla when compared to the LC group (p < 0.05).

The abundance of the Cyanobacteria, Elusimicrobia, Fibrobacteres, Lentisphaerae, Proteobacteria, and Spirochaetes phyla were lower (< 1%) in the P-20 group compared to the PC group (p < 0.05). Additionally, the abundance of the Bacteroidetes phylum was significantly higher in the P-80 group (p = 0.010), and in this group, Actinobacteria, Chloroflexi, SR1, Synergistetes, TM7, and WPS.2 phyla were not changed in their proportions when compared to those in the PC group (p > 0.05).

In contrast, samples of the L-20 group showed higher abundance of Firmicutes and Tenericutes phyla (p < 0.05) and only Chloroflexi, Cyanobacteria, SR1, Spirochaetes, Synergistetes, TM7, Verrucomicrobia, and WPS.2 phyla were unchanged in terms of their proportions when compared to those in the LC group (p > 0.05).

**Fig 4:**
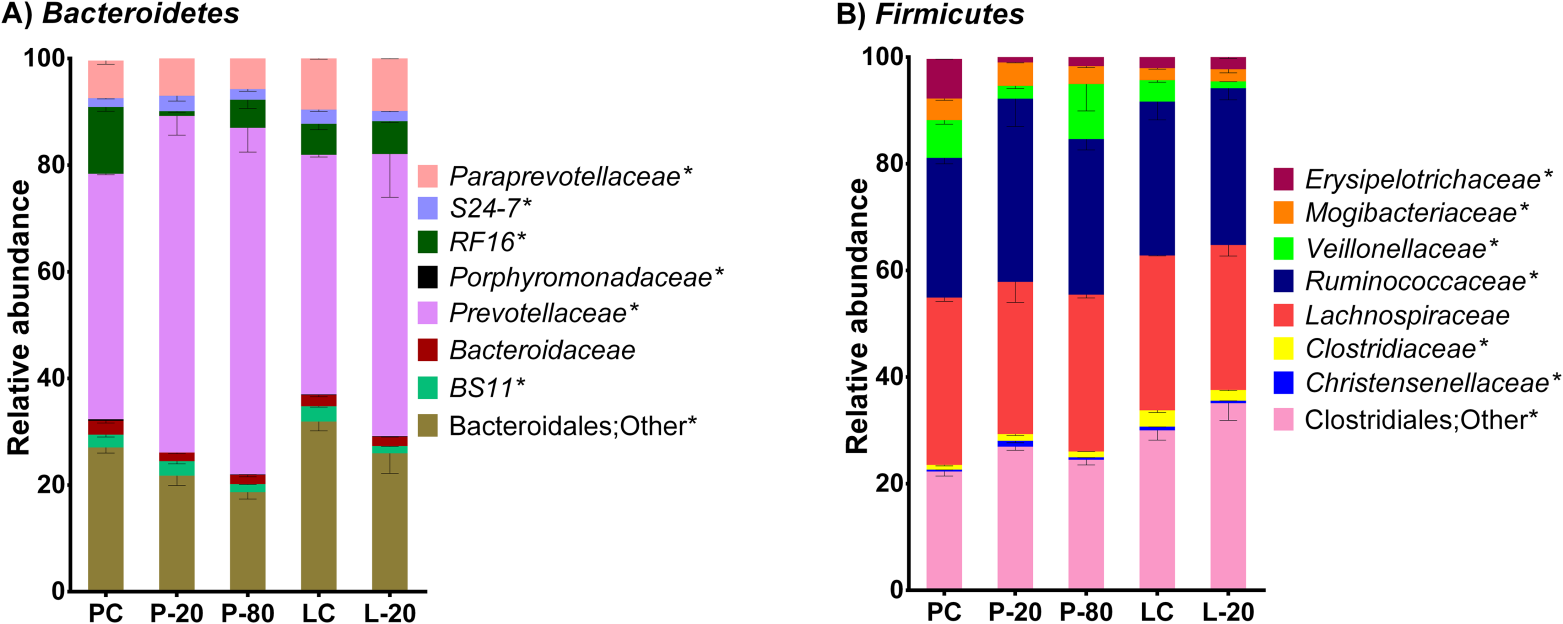
Abundance of operational taxonomic units (%) classified at the family level from (A) Bacteroidetes and (B) Firmicutes phyla, in pellet control (PC), pellet stored frozen at -20°C (P-20), pellet stored frozen at -80°C (P-80), lyophilized control (LC) and lyophilized sample stored frozen at -20°C (L-20) groups. * = statistical differences (P < 0.05) by Dunn’s test. n = 3 samples per group. P-20, P-80 and L-20 groups were stored during 12 months.

For the Firmicutes phylum, the Clostridium class was the most abundant, and a higher abundance was observed in all 12-month stored groups resulted, especially in the L-20 group (Table 2). At the family level, seven families were identified in the Firmicutes phylum (Fig 4B), and Ruminococcaceae, Lachnospiraceae, and Veillonellaceae, were the most abundant. Only the Lachnospiraceae family showed similar abundance in all groups sequenced (p = 0.071). There were no differences in the abundance of Ruminococcaceae when the rumen content was not stored (PC = 8.03% and LC = 9.74%). However, for both groups stored at -20°C higher abundance was observed when compared to that in the other groups (p = 0.019; L-20 = 14.67% and P-20 = 13.02%). Higher abundance of Ruminococcaceae was also observed in the P-80 group (10.31%), but only when compared to that in the PC group.

**Table 2:** Median and interquartile range of the of the abundance of operational taxonomic units classified at the class level in pellet control (PC), pellet stored frozen at -20°C (P-20), pellet stored frozen at -80°C (P-80), lyophilized control (LC) and lyophilized sample stored frozen at -20°C (L-20) group.

Pl	Class level	PC	P-20	P-80	LC	L-20	p-value
*B*	Bacteroidia	38.12 ± 1.3^d^	51.02 ± 1.4^b^	53.19 ± 1.1^a^	46.34 ± 1.5^c^	32.03 ± 1.7^e^	0.010
*C*	4C0d-2	2.82 ± 0.1^a^	0.08 ± 0.1^d^	0.93 ± 0.2^c^	2.23 ± 0.3^b^	2.17 ± 0.2^b^	0.012
*Fb*	Fibrobacteria	5.02 ± 0.3^a^	0.11 ± 0.1^e^	1.32 ± 0.3^d^	4.38 ± 0.3^b^	3.54 ± 0.5^c^	0.009
*Fi*	Bacilli	0.020 ± 0.0	0.040 ± 0.0	0.018 ± 0.0	0.017 ± 0.0	0.020 ± 0.0	0.134
*Fi*	Clostridia	28.51 ± 0.2^c^	37.32 ± 1.2^b^	34.63 ± 2.4^b^	30.09 ± 1.7^c^	47.81 ± 0.8^a^	0.011
*Fi*	Erysipelotrichi	2.29 ± 0.2^a^	0.63 ± 0.2^b^	0.73 ± 0.1^b^	0.82 ± 0.1^b^	1.19 ± 0.2^a^	0.021
*P*	α-proteobacteria	5.38 ± 0.9^a^	0.18 ± 0.1^e^	0.84 ± 0.2^d^	2.74 ± 0.3^b^	1.41 ± 0.3^c^	0.009
*P*	β-proteobacteria	0.06 ± 0.1	0.05 ± 0.0	0.02 ± 0.0	0.14 ± 0.2	0.09 ± 0.0	0.085
*P*	δ-proteobacteria	0.63 ± 0.1^a^	0.22 ± 0.1^b^	0.41 ± 0.1^a^^b^	0.20 ± 0.1^b^^c^	0.11 ± 0.0^c^	0.025
*P*	ε-proteobacteria	0.06 ± 0.0^a^	NI ^d^	0.01 ± 0.0^c^	0.03 ± 0.0^b^	0.01 ± 0.0^c^	0.010
*P*	γ-proteobacteria	0.40 ± 0.1^a^	0.02 ± 0.0^c^	0.13 ± 0.1^b^	0.31 ± 0.1^a^	0.12 ± 0.1^b^	0.017
*S*	MVP.15	0.19 ± 0.0^a^	0.05 ± 0.0^b^	0.05 ± 0.0^b^	0.06 ± 0.0^b^	0.04 ± 0.0^b^	0.050
*S*	Spirochaetes	3.69 ± 0.6^a^	0.62 ± 0.1^d^	1.24 ± 0.3^c^	2.04 ± 0.2^b^	2.08 ± 0.5^b^	0.015
*T*	Mollicutes	4.76 ± 0.4^b^	4.41 ± 0.7^b^^c^	3.76 ± 0.7^c^	4.93 ± 0.4^b^	6.43 ± 0.9^a^	0.031
*T*	RF3	0.27 ± 0.0^a^	0.05 ± 0.0^d^	0.09 ± 0.0^c^	0.09 ± 0.0^c^	0.19 ± 0.0^b^	0.014

Pl = phylum level, B = Bacteroidetes, C = Cyanobacteria, Fb = Fibrobacteres, Fi = Firmicutes, P = Proteobacteria, S = Spirochaetes, T = Tenericutes.

^a, b, c, d, e^ = values followed with superscript letters indicate statistical differences (p < 0.05) based on Dunn's test.

In addition, in the Proteobacteria phylum, the α-proteobacteria class was the most representative in all experimental groups. The PC group showed a higher proportion of this class when compared to that in the LC group (p = 0,009). However, the type of storage method resulted in a reduction in this class of bacteria, especially in the P-20 group, which resulted in the disappearance of ε proteobacteria class and the genus *Campylobacte*r, the only genus identified within this class.

In the Bacteroidetes phylum, six families were detected, and only Bacteroidaceae was not influenced by sample storage time and rumen sample storage method (P = 0.095; Fig 4A). However, the abundance of the Prevotellaceae family, the most abundant bacterial family in this phylum, was not changed when the rumen content was not stored (PC = 17.54% and LC = 20.80%), was higher in both pellet groups (P-20 = 31.60% and P-80 = 34.25), and was lower in the L-20 group (15.84%). In contrast, the Veillonellaceae family showed a lower abundance in both groups stored at -20°C (P = 0.034; L-20 = 0.63% and P-20: 0.90%) when compared to that in the PC (2.24%) and P-80 (3.65%) groups; however, its proportion in the LC group (1.41%) was lower only when compared to that in the P-80 group.

## Discussion

Understanding the influence of sample storage time and rumen sample storage method on ruminal bacterial composition has great importance to determine the ideal approach for studies on ruminal microbiology and for maximum comparability of results. In this study, three storage methods and four storage times were evaluated based on quality parameters and yield of metagenomic DNA extracted as well as the composition of the rumen bacterial community. Our results showed that the storage time of the ruminal sample can significantly reduce the yield of metagenomic DNA extracted. All storage methods showed differences in the rumen bacterial community proportions at the phylum, class, and family levels. Therefore, the hypothesis that the storage method and sample storage time could influence the yield and quality of metagenomic DNA obtained and modify the rumen bacterial composition was confirmed.

It is known that the predominant bacterial mass is adhered to ruminal fibrous particles [3, 36, 37]. Thus, to collect and conserve those particles would result in a high yield of metagenomic DNA in lyophilized samples. However, the procedure for obtaining the pellet was sufficient to remove microorganisms adhered to these particles resulting in a similar apparent metagenomic DNA yield in the PC and LC groups.

When the rumen bacterial community was studied, the PC group showed differences in bacterial proportions classified at the level of phylum, class, and family compared to those in the LC group. This difference is most clearly shown in principal coordinate analyses, and suggested that in the rumen samples, even without storage and similar yield and integrity DNA, the type of rumen sample processing before the extraction of metagenomic DNA can influence the final result of the rumen microbial population studies. Is plausible that these differences are due to the different detachment of different microbes from particles during the processing. This highlights the importance standardizing the processing method for rumen samples in microbiological labs and in the scientific community to allow for comparability of results.

Despite the similar apparent metagenomic DNA yield between methods at time 0 of storage, the high percentage of metagenomic DNA loss in the L-20 group during storage makes this method less suitable when ruminal samples must be stored for over three months. In addition, in this study, samples stored as L-20 showed a significant reduction in three of the most abundant bacterial phyla in the rumen, specifically, Bacteroidetes, Fibrobacteres, and Proteobacteria. In contrast, the lower OTU% values remaining after the pipeline for lyophilized groups suggest that this storage method results in a higher proportion of poorly sequenced passages and yields low quality or sequenced portions of very small size. Lyophilisation is widely used in the maintenance of different organisms; however, it is known that the steps comprising this process are capable of causing injury or cellular damage and changes in cell membrane permeability [38, 39]

The P-20 group resulted in a lower conservation efficiency because although it had metagenomic DNA loss lower than that of the L-20 group, P-20 resulted in a striking reduction in the proportion several bacterial phyla and the total disappearance of the ε proteobacteria class. These results indicate the low conservation efficiency of this storage method and the high sensitivity of these microorganisms to rumen sample storage. Freezer storage at -20°C is one of the most simple and inexpensive maintenance methods, which is based on a significant reduction in cell metabolism [40]. However, its disadvantage is that it appears to result in reduced viability of some microorganisms, due to damage to cells from the formation of ice crystals and the electrolytic variation in the temperature range used [39].

In contrast, despite the fact that the P-80 group was able to maintain metagenomic DNA yield for up to 6 months of storage, this group showed decreases in total number of OTU richness, as well as Chao 1, ACE, and Shannon Wiener indices when compared to those in the PC group. These variables are important measures that can be used to evaluate the efficiency of DNA extraction from different samples. Generally, more OTUs and higher richness and diversity indices could represent more species within samples [41].

In addition, our results of bacterial diversity using the Nellore bovine rumen showed high abundance of Bacteroidetes phylum, followed by the Firmicutes phylum. This finding was consistent with a previous study on the rumen of Zebu cattle [42, 43] and buffalo [44, 45]. However, in our study the storage of the rumen sample was able to change the proportion of these bacterial phyla, mainly in the L-20 group. These results, differ from those of Fliegerova et al. [12] wherein the storage temperature and the use of cryoprotectant additives did not influence the quantification of Firmicutes and Bacteroidetes.

In ruminants, Firmicutes is a relatively dominant rumen phylum, performing essential functions in energy conversion [40]. Previous studies have indicated that during metagenomic DNA extraction, these microorganisms are more resistant to mechanical lysis [6]. In our study, regardless of the storage method, there were increases in the proportions of this phylum in stored groups when compared to groups that did not undergo storage, which might indicate low sensitivity of the microorganism to sample storage. These results also suggest that when the rumen samples undergo storage before metagenomic DNA extraction, the abundance of the Firmicutes phylum, and specifically the Clostridia class, could be favoured.

Lachnospiraceae and Ruminococcaceae families are the most abundant in intestinal environments [46] and are highly specialized in the degradation of complex cellulose and hemicellulose of plant material [47]. Although they are considered phylogenetically similar, the Ruminococcaceae family was favoured by the storage of the rumen sample, whereas the Lachnospiraceae family was not affected. These results show that even though these microorganisms belong to the same phylum, these families have sensitivity differences to freezing per se and storage method. Thus, comparisons of results from studies in which the storage time and storage techniques of the ruminal samples are different should be done with caution.

Moreover, it is not advisable to store rumen samples when studying specific groups that show high sensitivity to storage, such as bacteria of the Fibrobacteres phylum; this microbial group is considered the most important phylum for the degradation of cellulosic plant biomass in the guts of herbivores [48] and has been greatly studied for its high industrial potential in the production of cellulases [49].

The lowest apparent yield of metagenomic DNA, assessed by fluorometry when compared to evaluation by spectrophotometry, can be due to the UV absorbance readings measuring molecules that absorb at 260 nm, including simple DNA and RNA tapes [50]. As the use of fluorophores results in the generation of a fluorescent signal upon binding to double-stranded DNA, such results will be more accurate than those obtained using methods employing a specific wavelength. Furthermore, the fluorescence can be deactivated by co-reagents or materials extracted from the rumen together with metagenomic DNA [6].

Additionally, the relative absorbance readings at A260/A280 nm and A260/A 230 nm have significance for most molecular biology techniques because they are used for the determination of metagenomic DNA purity [6, 50]. Our results showed that the purity of metagenomic DNA extracted after all storage methods and storage times was similar, suggesting that this variable is marginally affected by storage of the rumen sample.

## Conclusion

The results of this study suggest that the tested methods of storage for rumen contents influence the yield of extracted metagenomic DNA as well as results of analysis of the rumen bacterial community composition. The rumen sample storage time can significantly reduce the yield of metagenomic DNA. The storage method can influence the abundance of specific phyla, classes, and bacterial families studied in rumen samples. It can also affect the richness and diversity indices. Ruminal sample storage at temperatures of -20°C is not recommended because this method resulted in lower microbial conservation efficiency, which was demonstrated by the reduction or disappearance of some phyla and classes of bacteria in the sample. In addition, it must be emphasized that the comparison of results from studies in which time and storage techniques of ruminal samples are different should be avoided.

## Supporting information

S1 Fig DNA extracted integrity determined by agarose gel 0.5% from pellet control (PC), lyophilized control (LC), pellet stored frozen at -20°C during 3, 6 or 12 months, pellet stored frozen at -80°C during 3, 6 or 12 months, and lyophilized sample stored frozen at -20°C during 3, 6 or 12 months. (PNG)

S2 Fig Rarefaction curve indicating the number of operational taxonomic units based on the sequences obtained by sequencing the V4 region of 16S rRNA gene fragments from the samples. (PNG)

S1 Table Median and interquartile range of the abundance of operational taxonomic units (%) classified at the phylum level in the pellet control (PC), lyophilized control (LC), pellet stored frozen at -20°C (P-20), pellet stored frozen at -80°C (P-80), and lyophilized sample stored frozen at -20°C (L-20) groups. (DOCX)
