# Supplementary figures and images for: Studies on bacterial community composition are affected by the time and storage method of the rumen content
Source: PLoS One. 2017 Apr 28;12(4):e0176701. doi: 10.1371/journal.pone.0176701 (PMC5409139; doi:10.1371/journal.pone.0176701)

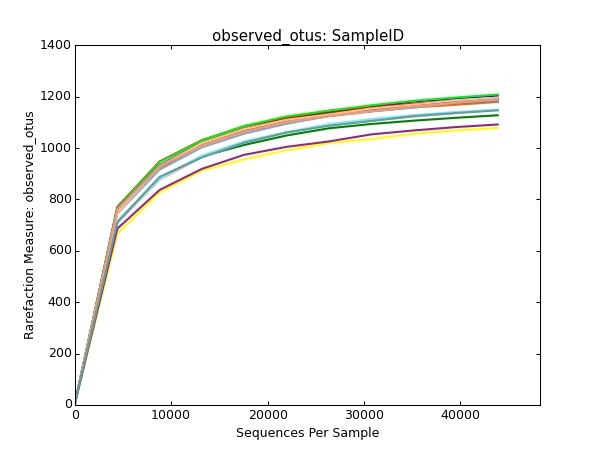

Supplement: S1 Fig — (PNG) [file pone.0176701.s001.png]

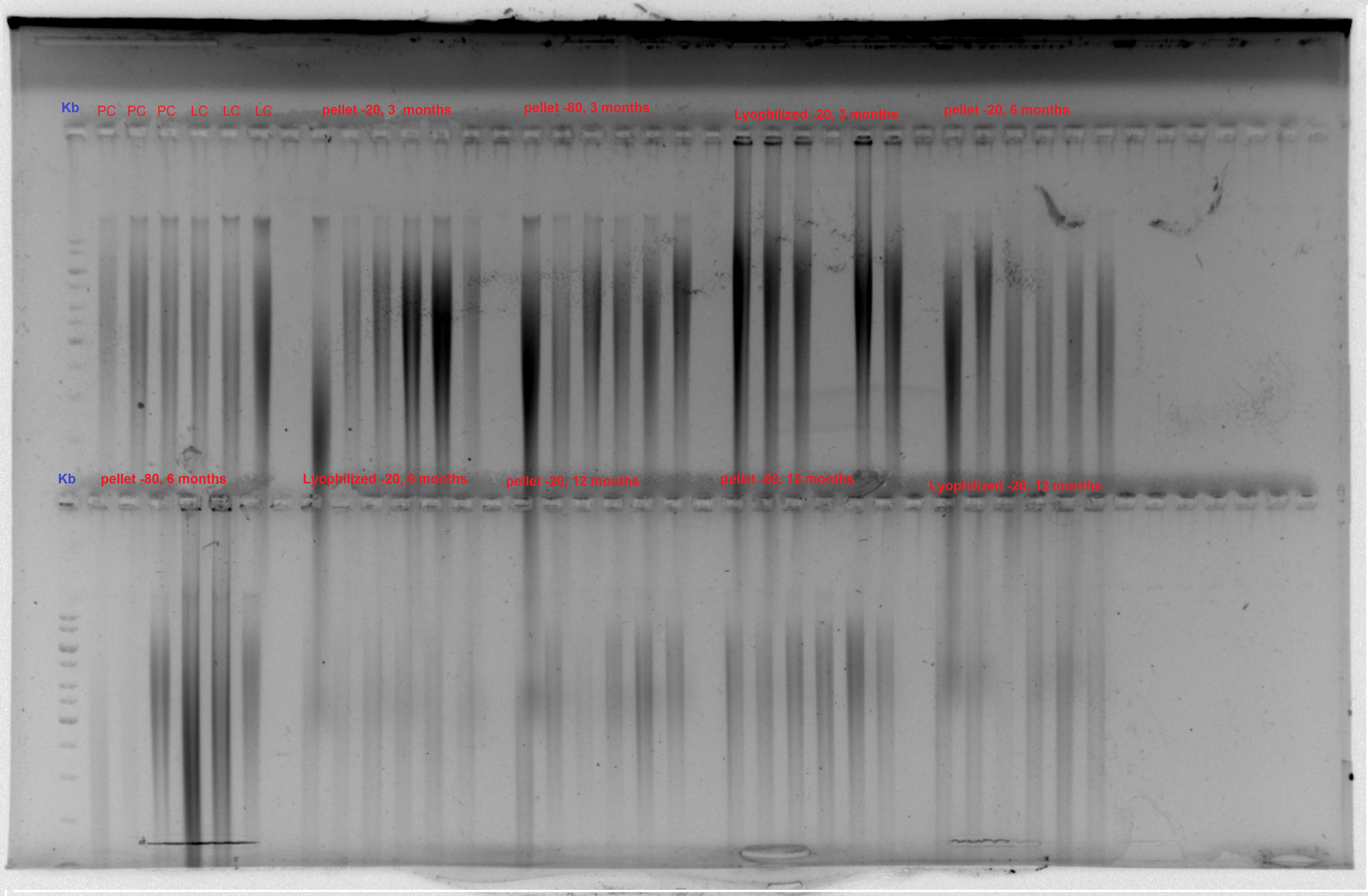

Supplement: S2 Fig — (PNG) [file pone.0176701.s002.png]
