# Supplementary material for: Studies on bacterial community composition are affected by the time and storage method of the rumen content
Source: PLoS One. 2017 Apr 28;12(4):e0176701. doi: 10.1371/journal.pone.0176701 (PMC5409139; doi:10.1371/journal.pone.0176701)
Supplement: S1 Table — (DOCX) [file pone.0176701.s003.docx]

| Phylum | PC | LC | P-20 | P-80 | L-20 | P-value |
| --- | --- | --- | --- | --- | --- | --- |
| *Actinobacteria* | 0,38±0,06^bc^ | 0,34±0,03^c^ | 0,62±0,05^a^ | 0,28±0,12^c^ | 0,43±0,06^a^ | 0,034 |
| *Bacteroidetes* | 38,12±1,32^d^ | 46,34±1,51^c^ | 51,02±1,37^b^ | 53,19±1,07^a^ | 32,03±1,67^e^ | 0,010 |
| *Chloroflexi* | 0,16±0,02^cd^ | 0,28±0,06^ab^ | 0,34±0,05^a^ | 0,11±0,02^d^ | 0,19±0,08^b^ | 0,024 |
| *Cyanobacteria* | 2,82±0,12^a^ | 2,23±0,33^b^ | 0,08±0,02^d^ | 0,93±0,24^c^ | 2,17±0,24^b^ | 0,012 |
| *Elusimicrobia* | 0,51±0,08^a^ | 0,14±0,05^ab^ | 0,04±0,01^d^ | 0,12±0,04^bc^ | 0,09±0,03^cd^ | 0,019 |
| *Fibrobacteres* | 5,02±0,32^a^ | 4,38±0,37^b^ | 0,11±0,05^e^ | 1,32±0,32^d^ | 3,54±0,50^c^ | 0,009 |
| *Firmicutes* | 30,83±0,44^d^ | 32,90±1,75^c^ | 37,87±1,07^b^ | 35,37±2,38^c^ | 48,81±0,64^a^ | 0,011 |
| *Lentisphaerae* | 0,43±0,06^a^ | 0,29±0,03^b^ | 0,06±0,02^d^ | 0,11±0,04^c^ | 0,13±0,06^c^ | 0,012 |
| *Planctomycetes* | 0,13±0,03^a^ | 0,06±0,02^ab^ | 0,09±0,01^a^ | 0,05±0,02^bc^ | 0,04±0,01^c^ | 0,043 |
| *Proteobacteria* | 6,51±0,78^a^ | 3,45±0,57^b^ | 0,47±0,21^e^ | 1,37±0,16d | 1,76±0,28^c^ | 0,009 |
| *SR1* | 0,79±0,11 | 0,39±0,05 | 0,46±0,20 | 0,47±0,26 | 0,41±0,11 | 0,202 |
| *Spirochaetes* | 3,88±0,61^a^ | 2,09±0,20^b^ | 0,62±0,06^d^ | 1,29±0,37^c^ | 2,12±0,51^b^ | 0,014 |
| *Synergistetes* | 0,07±0,01 | 0,07±0,01 | 0,05±0,03 | 0,05±0,01 | 0,04±0,01 | 0,097 |
| *TM7* | 0,14±0,02 | 0,05±0,03 | 0,16±0,05 | 0,07±0,03 | 0,14±0,11 | 0,092 |
| *Tenericutes* | 4,99±0,34^b^ | 5,02±0,35^b^ | 4,47±0,70^bc^ | 3,92±0,68^c^ | 6,57±0,97^a^ | 0,029 |
| *Verrucomicrobia* | 0,66±0,11^a^ | 0,60±0,11^ab^ | 0,25±0,01^c^ | 0,18±0,05^c^ | 0,57±0,03^b^ | 0,017 |
| *WPS.2* | 0,40±0,10^a^ | 0,19±0,03^b^ | 0,26±0,06^a^ | 0,28±0,02^a^ | 0,05±0,01^b^ | 0,020 |
| *Unclassified* | 1,78±0,11^a^ | 0,79±0,03^a^ | 0,38±0,08^c^ | 0,46±0,02^b^ | 0,43±0,10b^c^ | 0,016 |
